# Prognostic Value of Circulating Tumor Cells and Cancer Associated Macrophage-Like Cells in Metastatic Non-Small Cell Lung Cancer Patients: A Retrospective Exploratory Analysis

**DOI:** 10.32604/or.2025.069832

**Published:** 2026-01-19

**Authors:** Marco Siringo, Michela De Meo, Alain Jonathan Gelibter, Chiara Nicolazzo, Paola Gazzaniga

**Affiliations:** 1 Department of Molecular Medicine, Sapienza University of Rome, Rome, 00161, Italy; 2 Medical Oncology, Sant’Andrea University Hospital, Sapienza University of Rome, Via di Grottarossa 1035–1039, Rome, 00189, Italy; 3 Medical Oncology, Department of Radiological, Oncological and Pathological Science, Policlinico Umberto I, Sapienza University of Rome, Viale Regina Elena 324, Rome, 00161, Italy; 4 Department of Life Science, Health and Health Professions, Link Campus University, Via del Casale di San Pio V, Rome, 00165, Italy; 5 Department of Experimental Medicine, Sapienza University, Rome, 00161, Italy

**Keywords:** CellSearch®, circulating cancer-associated macrophage-like cells, circulating tumor cells, immunotherapy, non-small-cell lung cancer

## Abstract

**Objectives:**

Although immune checkpoint inhibitors (ICIs) and targeted therapies have reshaped treatment non-small cell lung cancer (NSCLC) paradigms, prognosis remains poor for many patients due to delayed diagnosis and resistance mechanisms. Liquid biopsy offers a minimally invasive approach to monitoring tumor evolution. Among circulating biomarkers, circulating tumor cells (CTCs) and cancer-associated macrophage-like cells (CAM-Ls) may provide complementary prognostic insights. The study aimed to evaluate the prognostic role of CTC and CAM-Ls dynamic in metastatic NSCLC patients.

**Methods:**

We retrospectively analyzed 77 patients with metastatic NSCLC who underwent CTC and CAM-L evaluation via the CellSearch® system at baseline (T0) and after three months of first-line treatment (T1) including chemotherapy, targeted therapy, or ICIs. Survival outcomes were analyzed using Kaplan-Meier and Cox regression analyses.

**Results:**

Conversion to CTC-negative status at T1 was associated with improved outcomes, with median overall survival (OS) and progression-free survival (PFS) of 33 and 18 months, respectively, vs. 10 and 6 months in persistently positive patients (both *p* < 0.001). CTC negativity at T1 remained an independent prognostic factor for OS (HR: 6.68) and PFS (HR: 5.91, both *p* < 0.0001). CAM-L positivity at T1 also correlated with longer OS (30 vs. 12 months) and PFS (13 vs. 6 months, both *p* < 0.0001), particularly among ICI-treated patients. Combined CTC and CAM-L assessment further refined risk stratification.

**Conclusions:**

Dynamic monitoring of CTCs and CAM-Ls provides actionable prognostic information in metastatic NSCLC. CTC-negative status predicted longer OS and PFS, while CAM-L positivity at T1 was associated with improved outcomes, particularly in ICI-treated patients. Combined assessment of both biomarkers may directly inform therapeutic decision-making, through early detection of outcomes.

## 1 Introduction

Non-Small Cell Lung Cancer (NSCLC) represents approximately 85% of all lung cancer cases and remains the leading cause of cancer-related mortality worldwide [1]. Advances in molecular biology and tumor genomics have identified actionable alterations, leading to targeted therapies that improved prognosis and quality of life in selected patients [2]. In parallel, immune checkpoint inhibitors (ICIs), particularly those targeting the PD-1/PD-L1 axis, have transformed treatment of oncogene-negative NSCLC and are now standard first-line options, either alone or in combination with chemotherapy, according to PD-L1 expression levels [3–5].

Despite significant advancements in the therapeutic landscape the prognosis for many NSCLC patients remains poor, largely due to late-stage diagnosis and the development of resistance mechanisms [6,7]. Consequently, there is a critical need for robust biomarkers that can guide therapeutic decision-making, enable real-time monitoring of treatment response, and predict disease progression.

In recent years, liquid biopsy has emerged as a minimally invasive approach capable of capturing the molecular and cellular heterogeneity of tumors through the analysis of circulating biomarkers, including circulating tumor DNA (ctDNA), circulating tumor cells (CTCs), extracellular vesicles (EVs) and microRNAs (miRNAs) [8,9].

Among these, CTCs have attracted particular attention due to their involvement in the metastatic cascade and their potential to offer a real-time snapshot of tumor evolution. Despite their promise, the clinical utility of CTCs in NSCLC is still limited by challenges in detection sensitivity, inter-platform variability, and the absence of standardized methodologies [10].

The CellSearch® system, the first FDA-approved platform for the enumeration of CTCs in certain metastatic malignancies, including breast, prostate, and colorectal cancers, has also demonstrated prognostic significance in NSCLC [11,12]. However, its broader clinical utility in NSCLC is limited due to inherently low CTC counts in many patients, variability in detection thresholds, and a lack of consensus on standardized cut-offs for clinical decision-making. These limitations contribute to inconsistent sensitivity and challenge the integration of CellSearch® into routine oncological workflows for NSCLC [13].

Notably, this system also enables the identification of additional blood-based biomarkers, including Cancer-Associated Macrophage-Like Cells (CAM-Ls), a recently described population of circulating myeloid cells characterized by their phagocytosis of tumor-derived material [14–16]. CAM-Ls have been identified across a range of malignancies and are notably absent in benign conditions, indicating potential diagnostic specificity. Furthermore, their presence, as well as morphological characteristics such as size and quantity, has been correlated with poor prognosis in several solid tumors. While preliminary data indicate that CAM-Ls may serve as indicators of treatment response, their clinical utility in NSCLC remains underexplored [17,18].

The aim of the present study to assess the potential for clinical utility of serial CTC and CAM-L enumeration as a tool for early treatment monitoring for populations defined by different first-line treatment type (ICIs vs. non-ICIs).

## 2 Materials and Methods

### 2.1 Study Population

One hundred twenty patients were consecutively enrolled at Policlinico Umberto I of Rome, Italy. All patients had a diagnosis of metastatic NSCLC. Tissue PD-L1 status, valuated by immunohistochemistry, and molecular profile, assessed through next generation sequencing (NGS), were determined according to Italian guidelines. The mandatory inclusion criteria were the availability of CTC and CAM-L counts both at baseline (T0) and after three months of first-line treatment (T1), diagnosis of metastatic NSCLC. We excluded patients who received previous systemic treatment, had concomitant tumors, or had incomplete clinical data. Detailed information can be found in Table 1.

**Table 1:** Baseline characteristics of the study population

Patients’ characteristics	No. (%)
**Age (years)**	
Median age (IQR)	67 (60–73)
**Sex**	
F	26 (34%)
M	51 (66%)
**Smoking status**	
No smoker	21 (27%)
Former smoker	35 (45%)
Current smoker	21 (27%)
**Histology**	
Adenocarcinoma Others	70 (91%) 7 (9%)
**Tissue PD-L1 status**	
≥50	15 (19%)
<50 or unknown	62 (81%)
**Sites of metastasis**	
1	28 (37%)
2	24 (31%)
≥3	25 (32%)
**First-line therapy**	
ICIs	30 (39%)
Chemotherapy Targeted therapy	31 (40%) 16 (21%)

Note: ICIs = Immune checkpoint inhibitors; PD-L1 = Programmed Death-Ligand 1; pts = patients; F = female; M = male.

Additional data collected included patient demographics, tumor histology, date of metastatic disease onset, number of metastatic sites, and treatment history.

Treatments received in the metastatic setting included chemotherapy, immunotherapy with ICIs and targeted therapy (EGFR and ALK inhibitors). Population was subclassified in two groups according to therapeutic treatment: ICIs and non-ICIs. ICIs population included patients without actionable alterations and PD-L1 status ≥50%, according to international guidelines.

Informed consent was obtained from all patients. This study was approved by the Institutional Review Board of Policlinico Umberto I of Rome (protocol n. 668/09, 09 July 2009; amended protocol 179/16, 01 March 2016) and is valis throughout the study period. The study was conducted in accordance with the Declaration of Helsinki.

### 2.2 CTC and CAM Categorizations

Patients were first categorized as CTC-negative or CTC-positive at baseline and at first follow-up (T1), using CTC ≥1 as cut-off for positivity, consistent with prior publications studies [18,19]. The same classification was performed on CAM-L, considering the cut-off of CAM-L ≥1 as positivity.

The first follow-up point (T1) was defined as 3 months ± 15 days after initiation of first-line treatment, allowing a 2-week window to accommodate scheduling variations and ensure comparability of results across patients.

All CTC and CAM-L analyses were performed in a single certified laboratory by operators blinded to patients’ clinical outcomes. To ensure consistency and reproducibility, quality control procedures included internal calibration, standardized processing protocols, repeated measurements for a subset of samples, standardized positive and negative control samples in each batch and verification of inter-operator consistency through repeated measurements on selected samples.

### 2.3 CTC and CAM-L Analysis Procedure

Peripheral blood (7.5 mL) was obtained from each patient at baseline and after three months of first-line treatment. Samples were drawn into CellSave tubes (Menarini Silicon Biosystems, Castel Maggiore, Italy), which contain EDTA and a cell-stabilizing fixative. Tubes were kept at room temperature and processed within 72 h in accordance with standardized laboratory procedures. No sample exceeded the 72-h processing window. These uniform collection and handling conditions ensured consistent preservation of cellular integrity and minimized technical variability.

Circulating tumor cells (CTCs) were quantified using the CellSearch® platform and the CellSearch® Epithelial Cell Kit (Menarini Silicon Biosystems). This system enriches epithelial cells through an anti-EpCAM–coated ferrofluid, followed by immunofluorescent staining for cytokeratins (CK), 4^′^,6-diamidino-2-phenylindole (DAPI) to visualize nuclei, and CD45 to exclude leukocytes. Cells were classified as CTCs if they exhibited round or oval morphology, contained a clearly identifiable nucleus, were CK-positive, and lacked CD45 expression.

Cancer-associated macrophage-like cells (CAM-Ls) were defined as CK+ and CD45+ cells measuring at least 30 μm, with one or more nuclei, an oblong or irregular contour, and a diffuse CK staining pattern (17, 20). CAM-L assessment was performed independently by two experienced operators blinded to clinical information. Any uncertain cases were jointly reviewed until consensus was reached.

Based on CTC assessments at both time points, patients were categorized into the four groups: neg/neg, neg/pos, pos/neg and pos/pos.

Furthermore, we performed a binary analysis by grouping patients based on their CTC at T0 and T1. Patients were classified into two main groups: those who were negative at T1 (including patients who were negative at both time points and those who converted from positive at T0 to negative at T1), and those who were positive at T1 (including patients who converted from negative to positive and those who remained positive at both time points). This dichotomization was adopted due to the limited sample size and to improve statistical power for analyses based on T1 CTC status. Among patients who were CTC-positive at T0, patients becoming CTC negative at T1 (CTC pos/neg) were termed CTC responders, while patients that remained positive at T1 (CTC pos/pos) were termed CTC non-responders. This classification was validated against clinical outcomes, including progression-free survival (PFS) and overall survival (OS), and analyses were stratified by treatment type.

CAM-L cells were assessed at baseline (T0) and at the first follow-up (T1). At T1, patients were categorized as CAM-L positive if at least one CAM-L cell was detected, and CAM-L negative otherwise. Dynamic changes in CAM-L (e.g., conversion from positive to negative) were not analyzed, as they were not considered relevant for this study. The cut-off for positivity was set at ≥1 cell. All CTC and CAM-L measurements were performed on the same blood specimen to ensure consistency and comparability.

### 2.4 Statistical Analysis

Continuous variables are reported as medians and ranges, whereas categorical variables are reported as counts and percentages. Continuous variables were analyzed as continuous, and their normality was assessed using the Shapiro–Wilk test. The primary objective of the study was to evaluate the prognostic significance of CTC dynamic changes (baseline to T1) in patients with metastatic NSCLC receiving first-line therapy. Secondary objectives included: evaluating the association of CAM-L positivity at T1 with clinical outcomes, exploring correlations between CTC and CAM-L status with different first-line treatments.

Survival analyses were performed in the overall population (patients with both CTC and CAM-L assessments at T0 and T1) and the defined subgroups according to treatment received (chemotherapy, ICIs and targeted therapy). Overall survival (OS) was defined as time from first-line treatment start to death from any cause; if a patient was not known to have died, survival time was censored at the last date the patient was known to be alive. Progression-free survival (PFS) was defined as the time from first-line treatment start to progression disease or death. Survival parameters were estimated based on the Kaplan-Meier method and summarized with medians and 95% confidence intervals (CI). In addition, univariate and multivariate Cox regressions were performed to obtain hazard ratio (HR) and 95% CI for death and progression disease. Multivariate models included clinically relevant covariates such as age, number of metastatic sites, treatment type, and tissue PD-L1 expression. The correlations between categorical variables were analyzed via chi-square tests and binary logistic regression. Missing data were minimal cases with missing values for a specific variable were excluded from analyses. Statistical significance was defined at *p* ≤ 0.05. Given the exploratory nature of this study, no formal correction for multiple testing was applied. All the statistical analyses were performed via IBM SPSS Statistics version 23 (IBM, Armonk, NY, USA).

## 3 Results

### 3.1 Patient Cohort, Tumor Subtypes and Baseline Characteristics

Individual patient data from 120 metastatic NSCLC patients treated between January 2019 and December 2024 were provided. A total of 43 patients were excluded because they did not meet the inclusion criteria. We selected 77 patients who received CellSearch CTC and CAM-L assessment at baseline (before starting first-line treatment), and at least one follow-up time after 3 months of first-line treatment.

The overall population was divided into three categories according to first-line treatment received. Thirty-one patients received immune checkpoint inhibitors (ICIs) alone or with chemotherapy according to PD-L1 status (15 patients receive ICIs alone because of PD-L1 expression ≥50% and the remaining received chemo + ICIs combination because of PD-L1 expression <50% or unknown.

Seventeen patients received targeted therapies because of oncogenic driver mutations detected on tissue samples through NGS: 11 were treated with EGFR inhibitors, 6 with ALK inhibitors. Chemotherapy treatment was platinum doublet in the remaining 30 patients, according to tumor histology (Table 1).

Clinical data of the overall population and distribution according to treatment received are resumed in Tables 2, A1 and A2.

**Table 2:** Grouping of patients based on CTC assessment at baseline and after three months of treatment with relative staging and treatment received

Patient groups according to CTC status	T0	T1	Stage (%)	*p*	Treatment (%)	*p*
Neg/neg: 21 pts	−	−	IVa 9 (42) IVb-c 12 (57)	0.480	ICis ± ChT 8 (38) Other treatments 13 (62)	0.921
Pos/neg 17 pts	+	−	IVa 7 (41) IVb-c 10 (59)	0.442	ICis ± ChT 11 (65) Other treatments 6 (35)	0.010
Neg/pos 10 pts	−	+	IVa 7 (70) IVb-c 3 (30)	0.162	ICis ± ChT 2 (20) Other treatments 8 (80)	0.193
Pos/pos 29 pts	+	+	IVa 15 (52) IVb-c 14 (48)	0.740	ICls ± ChT 9 (31) Other treatments 20 (69)	0.270

Note: ChT = chemotherapy; CTC = circulating tumor cells.

### 3.2 CTC and CAM-L at Baseline and First Follow-Up

In total, 46 patients had ≥1 CTCs at baseline and 40 at first follow-up, respectively. Overall, 21 patients had <1 CTC both at baseline and at first follow-up (neg/neg). In contrast, 10 patients had <1 CTC at baseline but ≥1 CTC at first follow-up (neg/pos); 17 patients converted from ≥1 CTC at baseline to <1 CTC at first follow-up (pos/neg) and 29 patients had ≥1 at both points (pos/pos). Meanwhile, 33 patients had ≥1 CAM-L at baseline and 37 patients at T1.

A total of 38 patients remained negative (<1 CAM-L) at both points, whereas 8 patients converted from negative at baseline to positive at T1. Additionally, 4 patients changed from positive at baseline to negative at T1, and 29 patients were positive at both time points (Table 3).

**Table 3:** CTC and CAM-L Positivity across time points and subgroups

Tumor Subgroup	N. pts	≥1 CTC Baseline (%)	≥1 CTC First Follow-Up (%)	≥1 CAM-L Baseline (%)	≥1 CAM-L First Follow-Up (%)
Overall	77	46 (60)	40 (52)	33 (43)	37 (48)
ICIs	30	20 (66)	12 (40)	13 (43)	24 (80)
Non-ICIs	47	26 (55)	28 (60)	20 (42)	13 (27)

Note: CAM-L = cancer-associated macrophage-like cells. N = number.

### 3.3 Survival Outcomes According to Serial CTC Level Status

**Figure 1:**
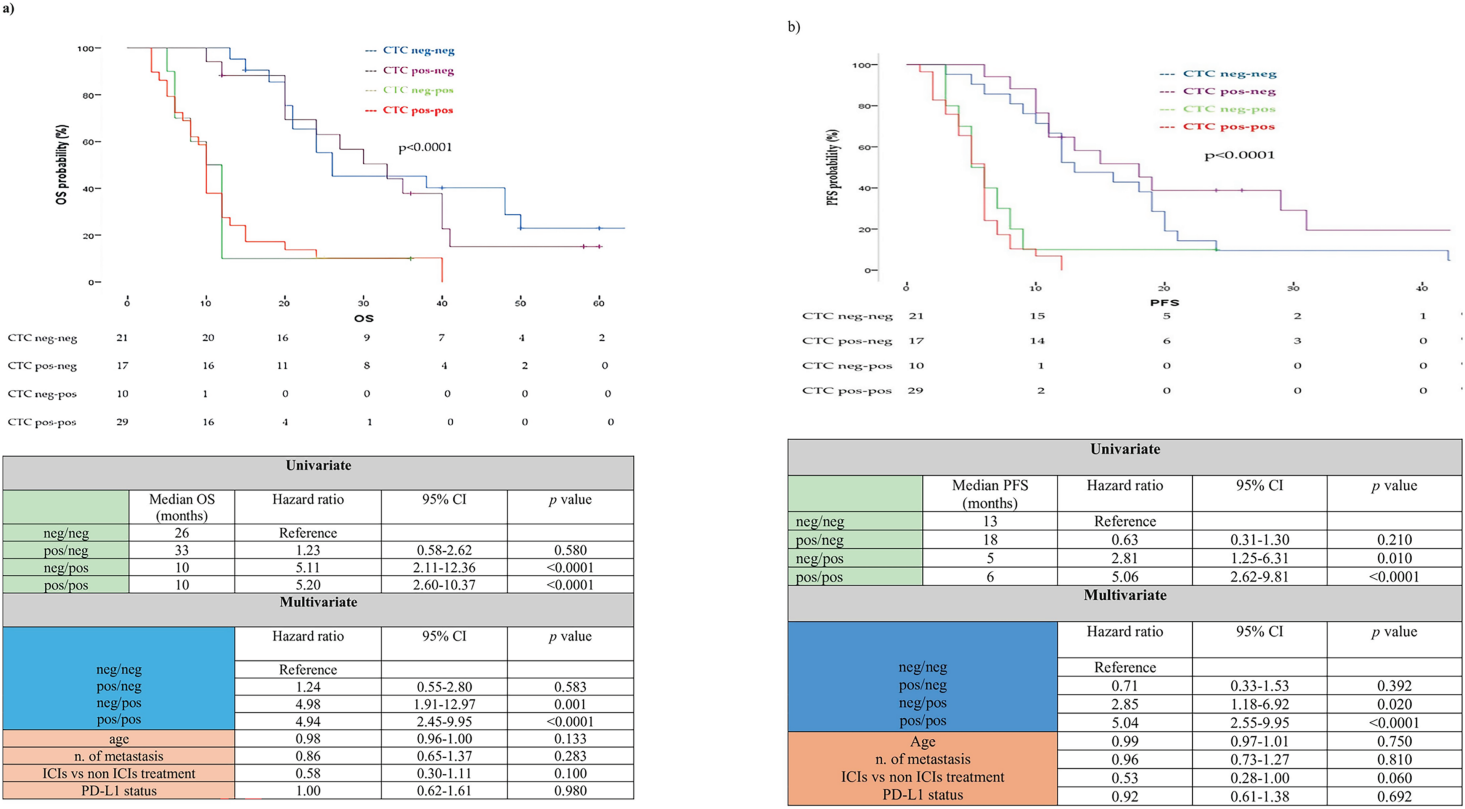
(**a**) Overall Survival (OS) and (**b**) Progression Free Survival (PFS) analysis of overall population according to circulating tumor cell (CTC) status at T0 and T1

As noted above, we identified four separate patient groups according to the status of CTC at T0 and T1: neg/neg, neg/pos, pos/neg, pos/pos. Using the cut-off of ≥1 CTC, OS among these four CTC change groups differed significantly (Fig. 1). Median OS for neg/neg, pos/neg, neg/pos, and pos/pos group was 26 months (95% CI 5.74–46.25), 33 months (95% CI 21.51–44.58), 10 months (95% CI 7.52–12.47) and 10 months (95% CI 9.14–10.85), respectively (*p* < 0.0001). Hazard ratios (reference group neg/neg) were 5.11 (95% CI 2.11–12.36) for the neg/pos group, 5.20 (95% CI 2.60–10.37) for the pos/pos group and 1.23 (95% CI 0.58–2.62) for the pos/neg group (Fig. 1a). Similarly, median PFS for neg/neg, pos/neg, neg/pos, and pos/pos group was 13 months (95% CI 8.51–17.48), 18 months (95% CI 10.48–25.51), 5 months (95% CI 2.93–7.06) and 6 months (95% CI 5.24–6.75), respectively (*p* < 0.0001). Hazard ratios (reference group neg/neg) were 2.81 (95% CI 1.25–6.31) for the neg/pos group, 5.06 (95% CI 2.62–9.81) for the pos/pos group and 0.63 (95% CI 0.31–1.30) for the pos/neg group (Fig. 1b).

**Figure 2:**
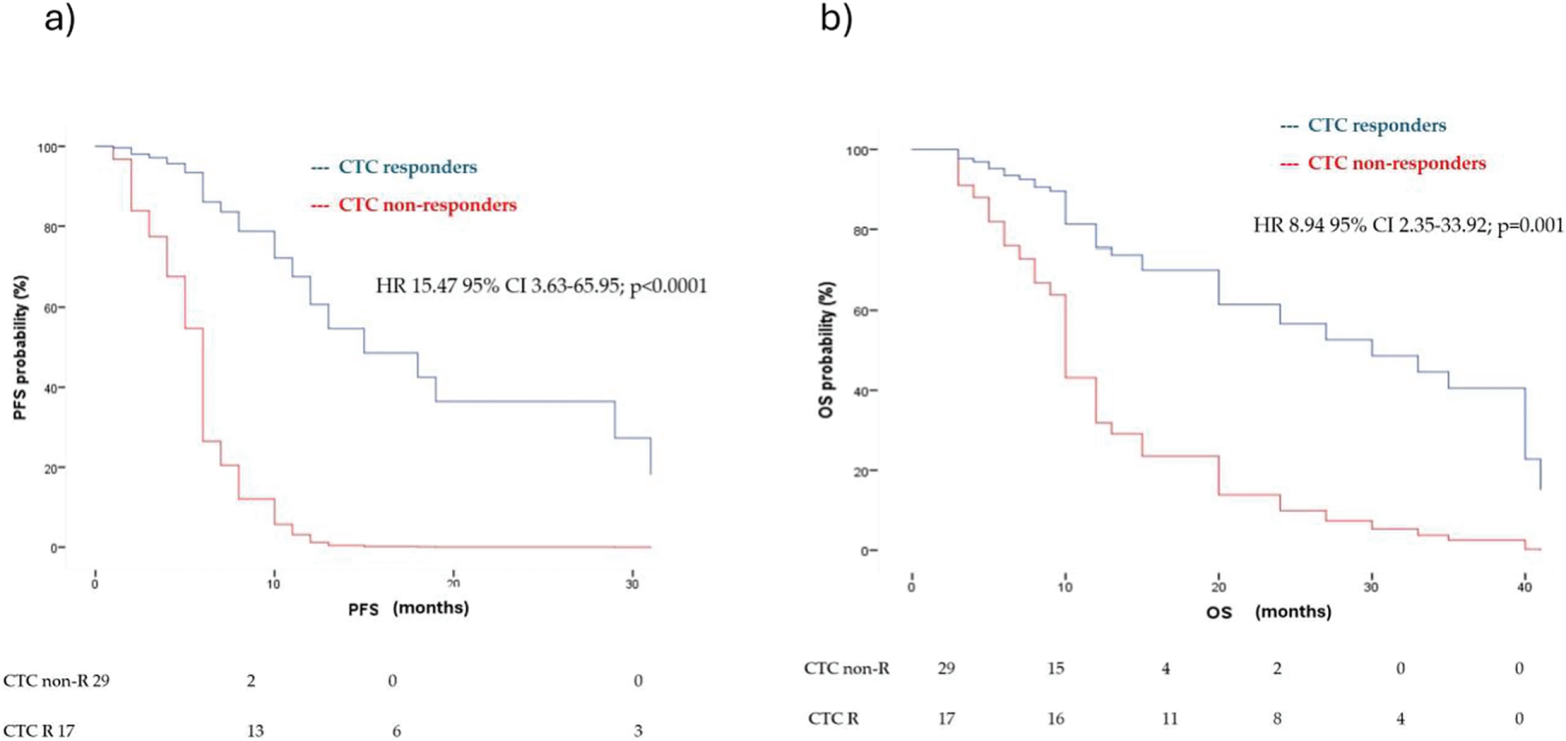
Multivariate analysis for (**a**) PFS and (**b**) OS in CTC responders (CTC pos/neg) and CTC non-responders (pos/pos) according to first-line treatment (immunotherapy vs. chemotherapy vs. target therapy), age, sites of metastasis and tissue Programmed Death-Ligand 1 (PD-L1) status. HR = hazard ratio, CI = confidence interval

Among patients who were CTC-positive at T0, CTC non-responders (pos/pos) had a significantly higher risk of disease progression compared to CTC responders (pos/neg), with a hazard ratio of 15.47 (95% CI: 3.63–65.95; *p* < 0.0001) (Fig. 2a). Similarly, CTC non-responders showed an increased risk of death relative to responders, with a hazard ratio of 8.94 (95% CI: 2.35–33.92; *p* = 0.001) (Fig. 2b).

**Figure 3:**
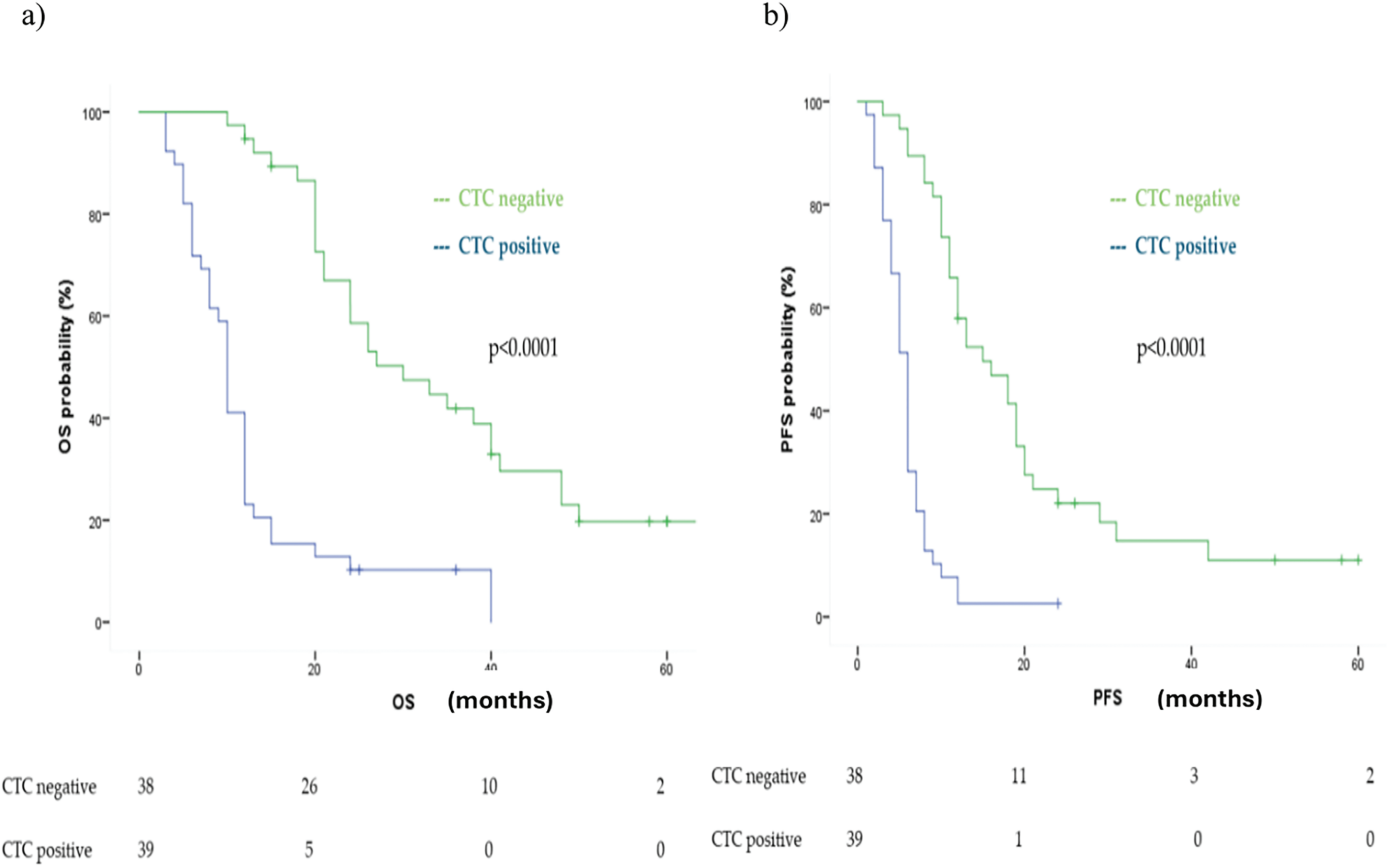
Kaplan Meier analysis of (**a**) OS and (**b**) PFS comparing CTC positive vs. CTC negative at first follow up (T1)

Furthermore, T1 CTC-negative patients (neg/neg, pos/neg) demonstrated significantly longer OS, with a median OS of 30 months (95% CI: 19.48–40.52), compared to T1 CTC-positive patients (pos/pos, neg/pos), who had a median OS of 10 months (95% CI: 9.14–10.86) (*p* < 0.0001) (Fig. 3a).

Similarly, PFS was significantly longer in the T1 CTC-negative group, with a median PFS of 15 months (95% CI: 9.19–20.80), compared to 6 months (95% CI: 5.26–6.73) in the CTC-positive group (*p* < 0.0001) (Fig. 3b).

Both findings were confirmed in multivariate analysis adjusted for age, number of metastatic sites, treatment type, and tissue PD-L1 expression, showing a HR for OS of 6.68 (95% CI: 2.72–16.44, *p* < 0.0001) and for PFS of 5.91 (95% CI: 2.58–13.54, *p* < 0.0001).

### 3.4 Survival Outcomes According to CAM-L Trends

CAM-L cells were assessed at baseline (T0) and at the first follow-up (T1). A sample was considered positive if at least one CAM-L cell was detected. Based on the T1 CAM-L status, patients were stratified into two groups.

**Figure 4:**
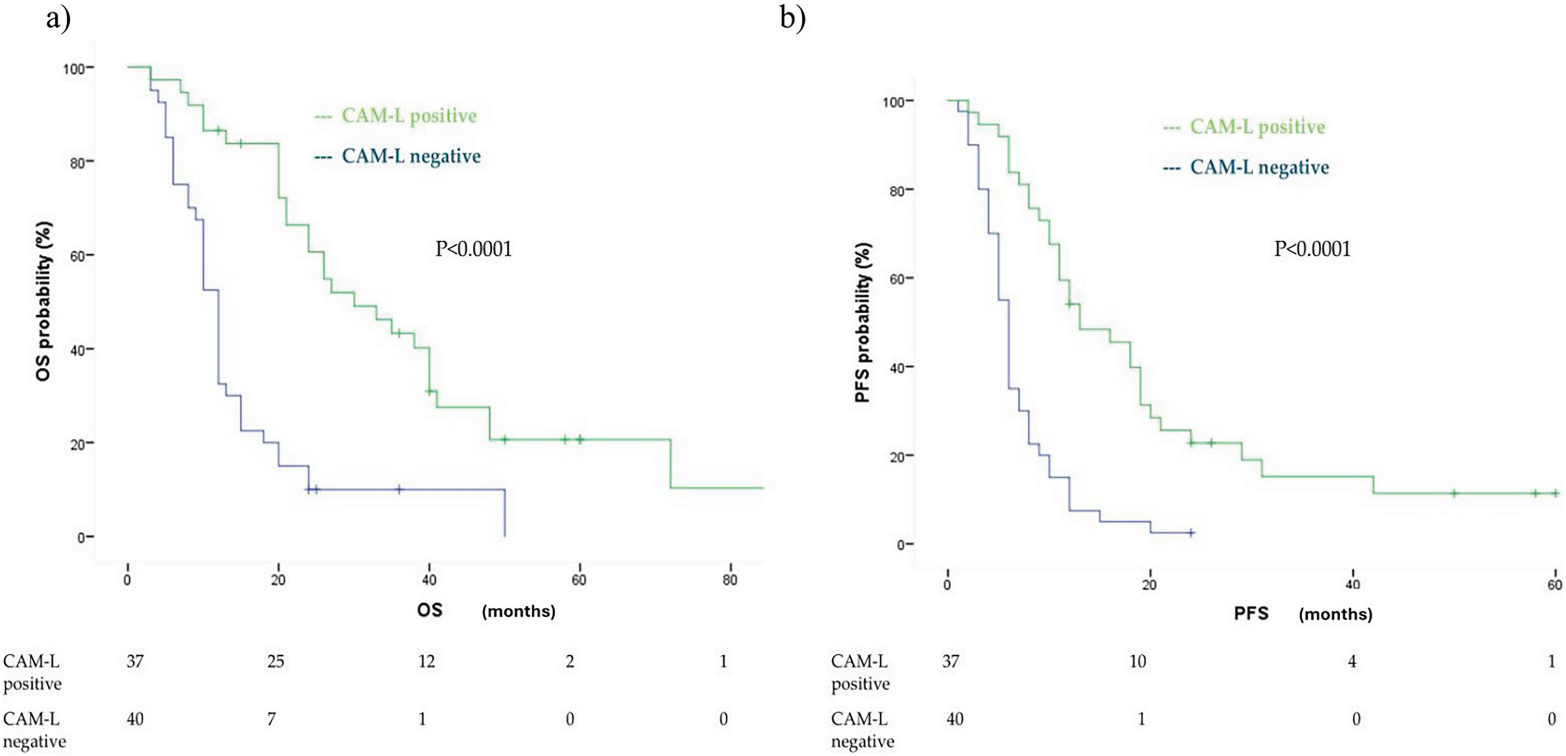
Kaplan Meier analysis of OS (**a**) and PFS (**b**) comparingcancer-associated macrophage-like cell (CAM-L) positive vs. CAM-L negative at first follow up (T1)

Unlike CTCs, T1 CAM-L positivity was significantly associated with improved median OS: 30 months (95% CI: 19.48–40.51) in CAM-L-positive patients, compared to 12 months (95% CI: 10.75–13.24) in CAM-L-negative patients (*p* < 0.0001). The corresponding HR was 0.28 (95% CI: 0.16–0.48, *p* < 0.0001). This association remained statistically significant in multivariate analysis adjusted for age, number of metastatic sites, treatment type, and PD-L1 expression (adjusted HR: 0.28, 95% CI: 0.12–0.62, *p* = 0.002) (Fig. 4a).

However, this association appeared to be influenced by the CTC status at T1, which showed a non-significant trend (HR: 0.59, 95% CI: 0.28–1.23, *p* = 0.16).

A similar pattern was observed for first-line PFS. Patients who were T1 CAM-L positive showed a longer median PFS of 13 months (95% CI: 7.22–18.77) compared to 6 months in T1 CAM-L-negative patients (95% CI: 5.26–6.74), with a highly significant difference (*p* < 0.0001) (Fig. 4b). The corresponding HR was 0.30 (95% CI: 0.18–0.50) in univariate analysis. This association was maintained after adjusting for age, number of metastatic sites, treatment type, and PD-L1 expression (adjusted HR: 0.23, 95% CI: 0.11–0.48, *p* < 0.0001), but was attenuated when accounting for T1 CTC status (HR: 0.72, 95% CI: 0.34–1.50, *p* = 0.38).

### 3.5 Survival Outcomes According to CTC Trends by First-Line Treatment Subgroups

**Figure A1:**
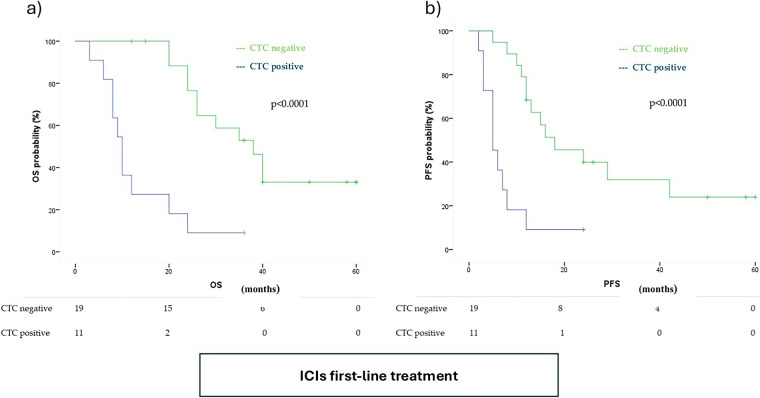
Kaplan Meyer of (**a**) Overall Survival (OS) and (**b**) Progression Free Survival (PFS) according to T1 CTC positivity in immune check point inhibitors (ICIs) population

As in the overall cohort, OS and PFS were compared between T1 CTC-negative and T1 CTC-positive patients within both ICIs and non-ICIs treatment subgroups. In the ICIs-treated subgroup, patients who were CTC-negative at T1 (including both neg/neg and pos/neg patterns) exhibited a significantly longer median OS of 38 months (95% CI: 28.65–47.34) compared to CTC-positive patients at T1 (neg/pos and pos/pos), who had a median OS of 10 months (95% CI: 7.91–12.08) (*p* < 0.0001). The corresponding univariate HR was 6.29 (95% CI: 2.37–16.72, *p* < 0.0001), which remained significant in multivariate analysis adjusted for age, number of metastatic sites, and PD-L1 status (HR: 8.17, 95% CI: 2.41–27.64, *p* = 0.001). Similarly, median PFS was significantly longer in T1 CTC-negative patients (18 months; 95% CI: 5.92–30.08) compared to T1 CTC-positive patients (5 months; 95% CI: 2.57–7.43) (*p* < 0.0001). The univariate HR for PFS was 4.44 (95% CI: 1.80–10.94, *p* = 0.001), and the multivariate HR was 5.25 (95% CI: 1.70–16.25, *p* = 0.004) (Fig. A1).

Similar findings were observed in the non-ICIs subgroup. T1 CTC-negative patients showed a significantly longer median OS of 21 months (95% CI: 15.31–26.68) compared to 10 months (95% CI: 7.95–12.05) in T1 CTC-positive patients (*p* < 0.0001). The univariate HR was 3.84 (95% CI: 1.89–7.80, *p* < 0.0001), and this was confirmed in multivariate analysis (HR: 3.84, 95% CI: 1.83–8.03, *p* < 0.0001). PFS analysis in the non-ICIs group confirmed these trends. T1 CTC-negative patients had a median PFS of 12 months (95% CI: 8.80–15.20), while T1 CTC-positive patients had a median PFS of 6 months (95% CI: 5.18–6.82), with a statistically significant difference (*p* < 0.0001). The univariate HR was 6.04 (95% CI: 2.73–13.38, *p* < 0.0001), and the multivariate HR was 7.63 (95% CI: 3.20–18.21, *p* < 0.0001). In summary, the HRs for death associated with CTC positivity were similar and statistically significant in both non-ICI (HR 3.84) and ICI (HR 4.17) groups, even after multivariate adjustment for age, number of metastases, and PD-L1 status.

### 3.6 Survival Outcomes According to CAM-L Trends by First-Line Treatment Subgroups

CAM-L trends were analyzed within both the ICIs-treated and non-ICIs-treated patient populations.

**Figure A2:**
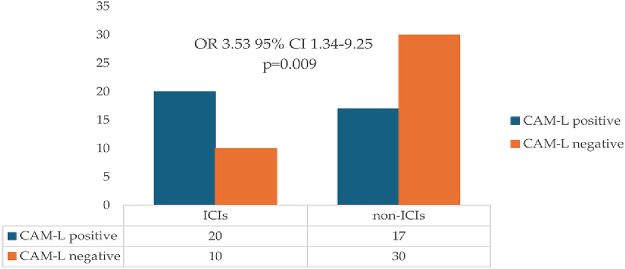
Distribution of cancer-associated macrophage-like cells (CAM-L) positivity in ICIs and non-ICIs populations

Patients receiving ICIs were significantly more likely to exhibit T1 CAM-L positivity, with an odds ratio (OR) of 3.53 (95% CI: 1.34–9.25, *p* = 0.009) (Fig. A2).

As expected, in the ICIs subgroup, T1 CAM-L positivity (neg/pos and pos/pos) was associated with markedly improved OS. Median OS in CAM-L-positive patients was 38 months (95% CI: 32.29–43.71), compared to 10 months (95% CI: 8.33–11.67) in CAM-L-negative patients (neg/neg and pos/neg), (*p* < 0.0001). The univariate HR was 0.08 (95% CI: 0.02–0.27, *p* < 0.0001), and this association remained significant in multivariate analysis adjusted for age, number of metastatic sites, and PD-L1 expression (adjusted HR: 0.07, 95% CI: 0.01–0.28, *p* < 0.0001).

A similar benefit was observed in terms of PFS. CAM-L-positive patients had a median PFS of 18 months (95% CI: 1.36–34.63), whereas CAM-L-negative patients had a median PFS of 5 months (95% CI: 3.32–6.67), (*p* < 0.0001). The univariate HR was 0.11 (95% CI: 0.03–0.34, *p* < 0.0001), and the multivariate HR was 0.10 (95% CI: 0.02–0.41, *p* < 0.0001).

**Figure 5:**
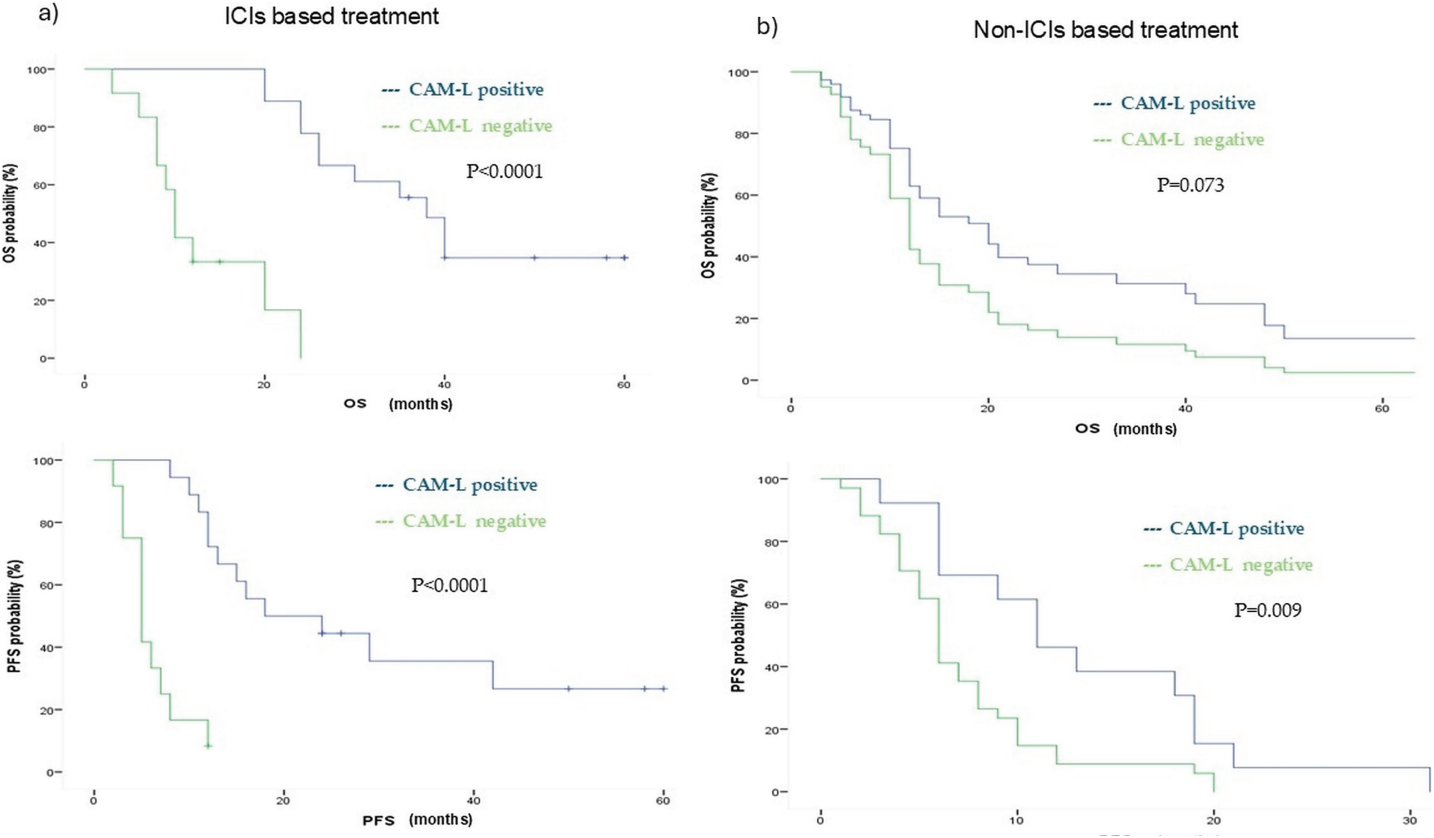
Kaplan Meyer showing ICIs (**a**) and non-ICIs (**b**) population OS and PFS outcomes according to CAM-L positivity. ICIs = Immune checkpoint inhibitors

In contrast, within the non-ICIs subgroup, no statistically significant difference in OS was observed between T1 CAM-L-positive patients (median OS: 21 months; 95% CI: 15.49–26.50) and CAM-L-negative patients (median OS: 12 months; 95% CI: 10.44–13.55), with an adjusted HR for age, number of metastasis, PD-L1 status of 0.54 (95% CI: 0.28–1.06; *p* = 0.073). However, CAM-L positivity was significantly associated with longer progression-free survival (median PFS: 11 vs. 6 months), with an adjusted HR for age and number of metastases of 0.41 (95% CI: 0.20–0.86; *p* = 0.019). (Fig. 5).

## 4 Discussion

This study provides valuable insights into the prognostic significance of CTCs and CAM-L in patients with metastatic NSCLC undergoing different first-line treatment regimens, including chemotherapy, ICIs, and targeted therapies. Our findings suggest that both CTC dynamics and CAM-L positivity provide robust prognostic information, which could help guide personalized treatment strategies, particularly in the context of immunotherapy.

In the context of NSCLC, establishing a reliable baseline cut-off value for CTCs is crucial for their clinical utility in prognosis and treatment monitoring. Although CTCs are rare in peripheral blood, their enumeration can provide meaningful insights into tumor dynamics, metastatic potential, and treatment efficacy. However, a standardized cut-off for CTC positivity in NSCLC has not been universally established, leading to variability in results across different studies and detection platforms [11,12,18]. Therefore, in our study we considered CTC ≥1 as cut-off for CTC positivity to maximize the number of patients that were CTC-positive according to relevant previous studies [19–22].

The concept of monitoring CTC dynamics over time has gained substantial interest in the context of cancer treatment, with several studies indicating that CTC count changes during therapy reflect treatment efficacy and tumor response [23,24]. Our study extends these findings by demonstrating that early reductions in CTC levels, especially within the first few months of treatment, are significantly associated with better OS and PFS in metastatic NSCLC. Specifically, patients who became CTC-negative at T1 exhibited significantly improved survival outcomes, which is consistent with previous studies highlighting the prognostic utility of CTCs as markers for treatment response and disease progression [25–28]. This emphasizes the importance of dynamic monitoring of CTCs during treatment, as a single point assessment may fail to capture the evolving tumor biology and the patient’s response to therapy.

Consistent with previous studies, CTC positivity correlated with worse OS across treatment subgroups. It is possible that CTCs play a dual role in the context of immunotherapy. On the one hand, higher CTC number could be associated with a higher burden of disease and poorer prognosis. On the other, CTCs may act as sources of tumor-associated antigens that can be recognized by the immune system, potentially enhancing antitumor responses in the presence of ICIs. In this context, the immunological effect of CTC biology warrants further mechanistic and clinical exploration [29].

While CTCs are well-established as prognostic markers, our study also underscores the potential role of CAM-L in assessing treatment outcomes. CAM-L positivity at T1 was strongly associated with improved OS and PFS, particularly in patients treated with ICIs. This result is consistent with previous studies suggesting that CAM-L, as a component of the tumor microenvironment, may provide additional prognostic value [18,30]. Our findings expand on show that CAM-L positivity, when combined with CTC data, may offer more robust predictive information, especially for patients receiving immunotherapy. The presence of CAM-L cells could indicate a more favorable immune response or better treatment efficacy, particularly with ICIs. However, this association was attenuated when adjusting for CTC status at T1, which suggests that the prognostic power of CAM-L may be influenced by the concurrent assessment of CTCs.

Importantly, the association between CAM-L positivity and improved OS was more pronounced in patients receiving ICIs than in those treated with chemotherapy or targeted therapies. This may reflect the role of tumor-associated macrophages in facilitating immune activation and enhancing the effects of checkpoint blockade. ICIs are known to modulate macrophage polarization toward an antitumor (M1-like) phenotype, which could explain the survival advantage observed in CAM-L–positive patients undergoing immunotherapy. This finding suggests that CAM-Ls may not only serve as prognostic markers but also reflect a favorable immune microenvironment that synergizes with ICIs [31].

The differential impact of CTC and CAM-L in the context of various treatments highlights the potential for personalized monitoring strategies.

While the findings of this study are promising, several limitations must be considered. First, the observational design and relatively small sample size limit the generalizability of the results. Additionally, although CTC and CAM-L assessments were conducted at baseline and after three months of treatment, a longer follow-up period would provide more robust data on the long-term prognostic value of these biomarkers.

Another limitation is the lack of a standardized cut-off value for CAM-L, along with the inability to distinguish between M1 and M2 macrophage polarization within CAM-Ls. The polarization of these macrophages may be influenced by the tumor microenvironment and treatment modality, especially ICIs. Notably, ICIs can promote M1 polarization, which is associated with an active immune response. To address this, incorporating a CD38 fluorescent-labeled antibody into the fourth channel of the CellSearch® system could enable more accurate differentiation of M1 and M2 macrophages. Alternatively, utilizing PD-L1 fluorescent-labeled antibodies in the fourth channel could offer valuable insights into the immune dynamics of CTCs and CAM-Ls, enhancing our understanding of their role in immunotherapy responses [32].

A further methodological limitation is that we applied binary thresholds for CTC and CAM-L positivity to align with prior CTC cut-off and to facilitate clinical interpretability. However, this approach may reduce statistical power compared to continuous modeling of biomarker counts. It is important to note that a subset of patients in this study underwent liquid biopsy analysis prior to the introduction of immunotherapy and chemotherapy combinations as first-line treatments for PD-L1 <50% tumors, which could have influenced the observed results.

Larger studies are warranted to validate the prognostic utility of CTCs and CAM-L and the possible added value of the CAM-L/CTC ratio, including those undergoing various treatment regimens, and to evaluate their potential to predict long-term clinical outcomes.

In the context of improving precision medicine in NSCLC, where ctDNA analysis can complement, and in some cases replace tissue biopsies for the detection of predictive biomarkers, the integrated assessment of CTCs and other blood-based analyses may provide valuable prognostic information and assist clinicians in monitoring disease progression [8,33]. In line with these objectives, our group is currently conducting a prospective, multicenter trial investigating the prognostic relevance of PD-L1–positive CTCs in patients with metastatic NSCLC receiving first-line immunotherapy, with or without chemotherapy through CellSearch.

## 5 Conclusion

In conclusion, this study highlights the prognostic significance of serial CTC and CAM-L assessments in metastatic NSCLC, with CTC dynamics providing important insights into treatment response and survival outcomes. Our results suggest that these biomarkers, may help stratify patients based on their likelihood of benefiting from different first-line treatments, including immunotherapy. Early biomarker monitoring may enable prediction of outcomes and support treatment escalation or de-escalation strategies. These findings highlight the potential of integrating CTC and CAM-L testing into clinical practice to advance personalized treatment in metastatic NSCLC. Given their potential to guide personalized treatment, the integration of CTC and CAM-L testing into clinical practice warrants further investigation.

## Data Availability

The data that support the findings of this study are available from the corresponding author, [Marco Siringo], upon reasonable request.

## Appendix

**Table A1:** The table resumes the overall population data including CTC and CAM-L positivity, the number and type of treatment received. Cht = chemotherapy, ICIs = Immune checkpoint inhibitors; ALKi = ALK inhibitors; EGFRi = EGFR inhibitors

Patients	CTC Group	CTC T0 Negative	CTC T1 Positive	First-Line Treatment	n. of Lines	CAM-L T1 Positive
1	Pos-pos	Positive	Positive	Platinum based Cht	2	Negative
2	Neg-neg	Negative	Negative	Platinum based Cht	2	Positive
3	Pos-pos	Positive	Positive	ICIs alone	1	Negative
4	Neg-neg	Negative	Negative	EGFRi	3	Positive
5	Pos-pos	Positive	Positive	Platinum based Cht	2	Positive
6	Neg-neg	Negative	Negative	EGFRi	3	Positive
7	Pos-neg	Positive	Negative	Platinum based Cht	2	Positive
8	Neg-pos	Negative	Positive	Platinum based Cht	2	Negative
9	Neg-neg	Negative	Negative	EGFRi	1	Positive
10	Neg-neg	Negative	Negative	Platinum based Cht	2	Positive
11	Pos-pos	Positive	Positive	Platinum based Cht	2	Negative
12	Pos-pos	Positive	Positive	Platinum based Cht	1	Negative
13	Neg-neg	Negative	Negative	Platinum based Cht	3	Negative
14	Neg-pos	Negative	Positive	Platinum based Cht	2	Negative
15	Neg-pos	Negative	Positive	Platinum based Cht	2	Positive
16	Pos-pos	Positive	Positive	EGFRi	1	Negative
17	Pos-pos	Positive	Positive	ICIs alone	1	Negative
18	Pos-pos	Positive	Positive	ICIs alone	1	Negative
19	Neg-pos	Negative	Positive	ICIs alone	1	Negative
20	Pos-pos	Positive	Positive	ALKi	1	Negative
21	Neg-neg	Negative	Negative	Platinum based Cht	2	Positive
22	Pos-pos	Positive	Positive	EGFRi	1	Negative
23	Pos-pos	Positive	Positive	ICIs alone	1	Negative
24	Neg-pos	Negative	Positive	ICIs + Cht	2	Negative
25	Neg-pos	Negative	Positive	Platinum based Cht	1	Negative
26	Pos-pos	Positive	Positive	EGFRi	3	Negative
27	Pos-neg	Positive	Negative	ICIs alone	1	Negative
28	Pos-pos	Positive	Positive	ALKi	2	Negative
29	Pos-neg	Positive	Negative	EGFRi	1	Positive
30	Neg-pos	Negative	Positive	EGFRi	2	Negative
31	Pos-pos	Positive	Positive	ICIs alone	1	Negative
32	Pos-pos	Positive	Positive	ALKi	2	Negative
33	Neg-neg	Negative	Negative	ICIs + Cht	2	Positive
34	Pos-neg	Positive	Negative	Platinum based Cht	2	Positive
35	Pos-pos	Positive	Positive	ALKi	1	Negative
36	Neg-neg	Negative	Negative	EGFRi	1	Negative
37	Pos-pos	Positive	Positive	Platinum based Cht	2	Negative
38	Neg-neg	Negative	Negative	ALKi	2	Positive
39	Pos-pos	Positive	Positive	ICIs alone	1	Negative
40	Neg-pos	Negative	Positive	Platinum based Cht	1	Negative
41	Pos-neg	Positive	Negative	ICIs alone	1	Positive
42	Pos-pos	Positive	Positive	EGFRi	3	Negative
43	Neg-neg	Negative	Negative	Platinum based Cht	2	Negative
44	Pos-neg	Positive	Negative	Platinum based Cht	2	Positive
45	Pos-pos	Positive	Positive	Platinum based Cht	2	Negative
46	Pos-neg	Positive	Negative	ICIs alone	1	Positive
47	Pos-pos	Positive	Positive	ICIs alone	1	Negative
48	Pos-neg	Positive	Negative	ICIs alone	1	Positive
49	Pos-neg	Positive	Negative	ICIs alone	1	Positive
50	Pos-neg	Positive	Negative	ICIs alone	1	Positive
51	Neg-neg	Negative	Negative	ICIs alone	1	Positive
52	Neg-neg	Negative	Negative	ICIs alone	1	Positive
53	Neg-neg	Negative	Negative	ICIs alone	1	Positive
54	Neg-neg	Negative	Negative	ICIs alone	2	Positive
55	Pos-neg	Positive	Negative	ICIs alone	2	Positive
56	Pos-neg	Positive	Negative	ICIs + Cht	1	Positive
57	Neg-neg	Negative	Negative	ICIs alone	1	Positive
58	Neg-neg	Negative	Negative	ICIs alone	1	Positive
59	Neg-neg	Negative	Negative	ICIs + Cht	2	Positive
60	Pos-neg	Positive	Negative	ICIs + Cht	1	Positive
61	Pos-pos	Positive	Positive	ICIs + Cht	2	Positive
62	Neg-pos	Negative	Positive	Platinum based Cht	2	Negative
63	Pos-neg	Positive	Negative	ALKi	1	Negative
64	Pos-neg	Positive	Negative	Platinum based Cht	2	Positive
65	Pos-pos	Positive	Positive	ICIs alone	1	Positive
66	Neg-neg	Negative	Negative	Platinum based Cht	2	Positive
67	Pos-pos	Positive	Positive	Platinum based Cht	2	Negative
68	Pos-pos	Positive	Positive	Platinum based Cht	1	Negative
69	Pos-pos	Positive	Positive	Platinum based Cht	1	Negative
70	Neg-pos	Negative	Positive	Platinum based Cht	1	Negative
71	Neg-neg	Negative	Negative	Platinum based Cht	2	Positive
72	Pos-pos	Positive	Positive	Platinum based Cht	1	Negative
73	Pos-neg	Positive	Negative	ICIs alone	1	Positive
74	Pos-neg	Positive	Negative	ICIs alone	1	Positive
75	Neg-neg	Negative	Negative	Platinum based Cht	2	Negative
76	Pos-pos	Positive	Positive	Platinum based Cht	1	Positive
77	Pos-pos	Positive	Positive	EGFRi	1	Negative

**Table A2:** Distribution of patients according to treatment received. ChT = chemotherapy, ICIs = Immune checkpoint inhibitors. R = responders; non-R = non responders

Group	Platinum-Based ChT	Targeted Therapy	ICIs	ICIs + ChT	*p*-Value
CTC neg/neg	8	5	6	2	0.30
CTC pos/neg	4	2	9	2	
CTC neg/pos	7	1	1	1	
CTC pos/pos	11	9	8	1	
T1 CAM-L pos vs. neg	8	18	5	12	0.02
T1 CTC pos vs. neg	18	12	10	7	0.27
CTC Response (R vs. non-R)	4	11	2	9	0.15
